# Minimal clinically-important differences for the “Liverpool Osteoarthritis in Dogs” (LOAD) and the “Canine Orthopedic Index” (COI) in dogs with osteoarthritis

**DOI:** 10.1371/journal.pone.0291881

**Published:** 2023-09-20

**Authors:** J. C. Alves, John F. Innes

**Affiliations:** 1 Divisão de Medicina Veterinária, Guarda Nacional Republicana (GNR), Lisbon, Portugal; 2 Faculty of Veterinary Medicine, Lusófona University, Lisbon, Portugal; 3 Centro de Ciência Animal e Veterinária, Lusófona University, Lisbon, Portugal; 4 MED–Mediterranean Institute for Agriculture, Environment and Development, Instituto de Investigação e Formação Avançada, Universidade de Évora, Pólo da Mitra, Évora, Portugal; 5 Movement Veterinary Referrals, Preston Brook, Runcorn, Cheshire, United Kingdom; 6 School of Veterinary Science, University of Liverpool, Leahurst Campus, Neston, Liverpool, United Kingdom; Shanghai Jiao Tong University Medical School Affiliated Ruijin Hospital, CHINA

## Abstract

**Objective:**

Osteoarthritis is the most common joint disease in companion animals. Several client-report outcome measures (CROMs) have been developed and validated to monitor patients and their response to treatment. However, estimates for minimal clinically-important differences for these CROMs in the context of osteoarthritis have not been published.

**Patients and methods:**

Data from the Clínica Veterinária de Cães (Portuguese Gendarmerie Canine Clinic) clinical records were extracted. Baseline and 30-day post-treatment follow-up data from 296 dogs treated for hip osteoarthritis were categorized based on an anchor question, and estimates of minimal clinically-important differences (MCIDs) using distribution-based and anchor-based methods were performed.

**Results:**

For the LOAD, the anchor-based methods provided a MCID estimate range of -2.5 to -9.1 and the distribution-based methods from 1.6 to 4.2. For the COI, the anchor-based methods provided a MCID estimate range of -4.5 to -16.6 and the distribution-based methods from 2.3 to 2.4. For the dimensions of COI, values varied from -0.5 to -4.9 with the anchor-based methods and from 0.6 to 2.7 with the distribution-based methods. Receiver operator characteristic curves provided areas under the curve >0.7 for the COI, indicating an acceptable cut-off point, and >0.8 for the LOAD, indicating an excellent cut-off point.

**Conclusion:**

Our estimates of MCIDs for dogs with OA were consistent with previously proposed values of -4 for the LOAD and -14 for the COI in a post-surgical intervention context. ROC curve data suggest that LOAD may more reliably differentiate between anchor groups. We also presented estimates from COI of -4 for Stiffness, Function, and Gait and -3 for quality of life. These estimates can be used for research and patient monitoring.

## Introduction

Osteoarthritis (OA) is a commonly diagnosed disease in veterinary medicine, affecting an expected growing number of animals worldwide [1, 2]. It has been associated with 80% of all lameness diagnoses in dogs [3]. For that reason, there is a need for proper evaluation tools to assess these patients. While objective modalities, such as gait analysis or weight-bearing evaluation, are accurate and reliable [4, 5], they are not widely available. Following a similar trend in human medicine, different client-reported outcome measures (CROM) have been developed and introduced in veterinary medicine over the last few decades [6, 7]. This patient-centered approach allows for a complete assessment of the multi-dimensional construct of OA and OA-related pain [8, 9].

While the patient reports on their experience in human medicine, in veterinary medicine, the client is the most common proxy. In addition to being able to identify changes and degrees of their pet’s subjective status, owners can also interpret changes over an extended period of time [10, 11]. Several CROMs have been developed to assess canine joint disease, including the Liverpool Osteoarthritis in Dogs (LOAD) [12, 13] and the Canine Orthopaedic Index (COI) [14]. Both have been validated in original language and translated versions [15, 16]. They have also been recommended for use in dogs with OA in a recent COSMIN-based systemic review [17] and for chronic pain in WSAVA guidelines [18].

In addition to being validated, estimates of specific minimal clinically-important differences (MCID) should be determined for individual CROMs. Having this set threshold allows for the success of a given treatment in individual patients [19] and for study design and sample size estimates in research and clinical trials [20]. Anchor-based methods or distribution-based methods can be used to estimate MCID. The first compares changes in CROMs to an explicit metric of the proxy’s opinion of the change. In contrast, the second relies on the statistical characteristics of a group’s baseline scores to determine how much of a change would be clinically important [21]. The evaluation of MCID also has to be done under clinical contexts.

This study aimed to estimate MCIDs for LOAD and COI in dogs with osteoarthritis.

## Materials and methods

The study protocol was approved by the ethical review committee of the University of Évora (Órgão Responsável pelo Bem-estar dos Animais da Universidade de Évora, approval n° GD/37187/2021/P1) and complies with relevant institutional, national and ARRIVE guidelines for the care and use of animals. Written, informed consent was obtained from the Institution responsible for the animals (Republican National Guard, Portugal).

Data were obtained from the clinical information of dogs presented for treatment for bilateral hip OA at the Clínica Veterinária de Cães (Portuguese Gendarmerie Canine Clinic). To be included in the study, dogs had to be diagnosed with bilateral hip OA, have available radiographic results consistent with the disease, and have confirmed absence of any other disease. As a standard approach the Clínica Veterinária de Cães Before treatment and at set intervals, the canine handlers complete an online version of the LOAD, the COI, and an anchor question. The Portuguese versions of the LOAD and COI have been previously validated [15, 16, 22]. Only patients with a single treatment modality were considered (e.g., a nonsteroidal anti-inflammatory drug or an intra-articular platelet-rich plasma administration). The anchor question was “How do you describe your dog’s overall quality of life”. The possible responses were “Poor”, “Fair”, “Good”, “Very Good”, and “Excellent”. Only the pre-treatment (T0) and the 30 days post-treatment (+30d) data were used in this study. Only patients with T0, +30d, and anchor question answers were included.

Relevant clinical data were exported to an Excel (Microsoft, Seattle, USA) spreadsheet. Statistical analyses were performed with commercially available software (IBM SPSS Statistics version 20). For the subsequent analysis, two groups were set: a “the same” and a “somewhat better” groups, based on the canine handler’s responses to the anchor question at +30d. The “the same” group comprised the animals with the same response at the T0 and +30d evaluations. The “somewhat better” group included the animals where a one-level better response was obtained at the +30d evaluation compared to the T0 evaluation. Baseline characteristics of the two groups were compared with the Mann-Whitney U test, while categorical data were compared with Fischer’s exact test. The Wilcoxon signed-rank test was used to compare changes in scores from T0 to +30d. The Mann-Whitney U test was used to compare differences in the CROMs and the mean change in the CROMs between the groups. Significance was set at p < 0.05.

We used four anchor-based methods to calculate the MCID. First, the “average change” (AC) was determined, corresponding to the mean change in the score of the ‘somewhat better’ group. A “change difference” was also determined, defined as the difference in the average change in score between the “somewhat better” and “the same” groups. The third method consisted in calculating the “minimum detectable change” (MDC). The MDC is the smallest change that can be considered beyond the measurement error at a 95% confidence level. Since an improvement with the LOAD and COI consisted of a reduction in total score, the MCID was equal to the lower value of the 95% confidence interval for the average change in the “the same” group’s score. Finally, a receiver operating characteristic (ROC) curve was used to define the point that best discriminated between the two groups. This optimal cut-off point was estimated using the point that maximized specificity and sensitivity. The area under the ROC curve (AUC) was also calculated to assess reliability. Based on previous reports, AUC values between 0.7 and 0.8 were considered acceptable, and 0.8 and 0.9 were considered excellent.

In addition to the anchor-based methods, two distribution methods were used to estimate the MCID. One was based on the effect size, calculated as the difference in mean score from T0 to +30d (in the present example) divided by the standard deviation (SD) of the T0 scores. Since an effect size of 0.2 is considered small, MCID with the following formula: (SD_T0_*0.2) [21]. The second method was based on the “standard error of measurement” (SEM) of CROM scores since SEM is an intrinsic property of the CROM and, therefore, independent of the patient cohort considered [21]. SEM with the following formula: SEM = SD* √ (1-r). In this case, “r” is the reliability of the instrument. For LOAD, a previously published value was used [23]. For the COI and its dimensions, previously calculated “r” values were used [16].

## Results

Data from 296 animals fulfilled the inclusion criteria. The breeds represented were German Shepherd Dogs (n = 160), Labrador Retriever (n = 56), Belgian Malinois Shepherd Dogs (n = 45), Dutch Shepherd Dog (n = 23), Rottweiller (n = 3), and others (n = 9). One hundred eighty were intact males and 116 were intact females, with a mean age of 7.8±2.1 years and a body weight of 30.3±6.2kg. Forty two had mild hip OA (14.2%), 162 had moderate hip OA (54.7%), and 92 had severe hip OA (31.1%) according to the Orthopedic Foundation for Animals hip grading scheme. Different treatments were identified: nonsteroidal anti-inflammatory drugs (meloxicam n = 8 and carprofen n = 8), intra-articular treatments (hyaluronan n = 35, triamcinolone n = 36, and hyaluronan+triamcinolone n = 33), photobiomodulation (n = 23), mesotherapy (n = 62) and biologicals (platelet rich plasma n = 53 and blood cell secretome, n = 38).

The mean T0 and +30d scores for LOAD and COI are presented in Table 1. Both CROMs demonstrated a significant difference between pre-treatment and post-treatment scores.

**Table 1:** Mean and standard deviation (SD) pre-treatment and 30 after-treatment scores for Liverpool Osteoarthritis in Dogs (LOAD) and Canine Orthopedic Index (COI).

CROM		n	T0		+30d		p value
			mean	SD	mean	SD	
LOAD (0–52)		296	21	10	15	12	<0.01*
COI	Stiffness (0–16)	296	6	3	4	4	0.04*
	Function (0–16)		6	4	4	4	0.03*
	Gait (0–20)		9	5	6	5	0.04*
	QOL (0–12)		6	3	4	3	0.01*
	Overall (0–64)		28	14	19	4	<0.01*

QOL–Quality of Life.

* indicates significance.

Considering the anchor question, there were 152 (51.4%) dogs in the “somewhat better” and 144 (48.6%) in the “the same” group. Scores at T0 and +30d for these two groups are presented in Table 2.

**Table 2:** Scores in the “the same” and “somewhat better” groups at T0 and +30d.

CROM		"The same" group		"Somewhat better" group		P value
		mean	SD	mean	SD	
**LOAD**				** **	** **	
	n	152		144		
	T0	18.05	0.96	19.85	10.07	0.054
	30d	20.33	0.9	11.26	9.87	0.005*
	mean change	2.28		-8.59		0.027*
**COI**						
	**Stiffness**					
	n	152		144		
	T0	5.09	0.91	6.08	3.37	0.061
	30d	6.19	1.07	2.04	3.43	0.011*
	mean change	1.1		-4.04		0.029*
	**Function**					
	n	152		144		
	T0	5.17	1.1	6.25	4.13	0.731
	30d	6.36	1.11	2.11	3.93	0.006*
	mean change	1.19		-4.14		0.078*
	**Gait**					
	n	152		144		
	T0	7.58	1.11	9.25	4.72	0.147
	30d	9.38	1.07	4.51	4.89	0.009*
	mean change	1.8		-4.74		0.014*
	**QOL**					
	n	152		144		
	T0	5.04	5.04	6.21	2.76	0.147
	30d	6.29	0.18	3.00	2.68	0.017*
	mean change	1.25		-3.21		0.002*
	**Overall**					
	n	152		144		
	T0	22.88	1.34	27.79	14.07	0.271
	30d	28.21	0.93	11.66	14.14	0.011*
	mean change	5.33		-16.13		0.037*

COI—Canine Orthopedic Index; LOAD—Liverpool Osteoarthritis in Dogs; QOL–Quality of Life.

* indicates significance.

The MCID estimates with the four anchor-based methods and the two distribution-based methods are shown in Table 3.

**Table 3:** MCIDs for Liverpool Osteoarthritis in Dogs and Canine Orthopedic Index.

		Anchor-based				Distribution-based	
CROM		AC	CD	MDC	ROC Curve (AUC)	Effect size	SEM
LOAD		-8.6	-9.1	-4.4	-2.5 (0.872)	±1.6	±4.2
COI	Stiffness	-4.0	-4.2	-1.2	-1.0 (0.777)	±0.7	±1.1
	Function	-4.1	-4.3	-1.4	-0.5 (0.774)	±0.8	±1.6
	Gait	-4.7	-4.9	-2.0	-1.5 (0.770)	±1.1	±2.7
	QOL	-3.2	-3.3	-1.4	-0.5 (0.768)	±0.6	±1.8
	Overall	-16.1	-16.6	-14.5	-4.5 (0.778)	±2.4	±2.3

AC–Average Change; AUC–Area under the curve; CD–Change difference; MDC–Minimum detectable change; ROC—Receiver operating characteristic; SEM—standard error of measurement.

The four anchor-based methods provided a range of MCIDs for each CROM (2.5 to 9.1 for LOAD and -4.5 to 16.1 for COI). Different ranges were also provided for the dimensions of COI. In the two different distribution-based methods, the MCID for LOAD ranged from 1.6 (effect size) to 4.2 (SEM), while the MCID for COI ranged from 2.3 (SEM) to 2.4 (effect size), showing a variation depending on the method applied. All AUCs calculated by the ROC curve were greater than 0.7, indicating an acceptable cut-off point. The greater AUC was found with LOAD (0.867) and the smallest with the quality of life dimension of the COI (0.770).

## Discussion

In the presented study, we estimated MCIDs for the LOAD and the COI, both validated for use in dogs with OA. We used data available from a population of police working dogs submitted to various treatments. The +30d follow-up moment was selected based on the expectation that it would be enough to obtain different responses to treatment, spreading answers to the anchor question and allowing us to estimate the MCID. However, this should not be considered a recommendation for evaluation post-treatment outcome in dogs with OA, as most treatments are expected to produce an earlier effect. Since the LOAD and the COI are a part of the routine follow-up for this population of dogs, and all fields are mandatory in the follow-up, we obtained results for both CROMs in all patients. The baseline values for the LOAD and COI were similar to the ones previously published for police working dogs with OA [24–31].

It is well established that the estimates of MCIDs can be affected by extrinsic and intrinsic factors [32]. While the present study provided results in an important clinical context, as is OA, it is important to remember that the patients included comprise a very homogenous sample, as the animals represent a set number of breeds and experience similar housing, feeding, and exercise conditions. Future studies will be needed to evaluate if this context influences the results. Still, the obtained results are very similar to a previous report, estimating MCIDs for the same CROMs in dogs surgically treated for cruciate ligament disease [20].

We followed a variety of methods to generate MCIDs. It has been argued that anchor-based estimates are more clinically-relevant, but distribution methods are based on larger data set [33]. With distribution methods, the SEM is preferred, as it is an intrinsic property of the CROM [21]. The four anchor-based methods we used have been previously used in human [34] and animal patients [20]. Similar to those studies, we observed that the different methods generated different MCID estimates. The largest estimate for the LOAD was for “change difference” at -9.1, as for the COI, at -16.6. For the various dimensions of the COI, the largest estimates were also obtained with “change difference”, ranging from -3.3 (for quality of life) to -4.9 (with gait). On the opposite end, the ROC curve generated the lowest estimates. The AUC of the ROC curve showed an acceptable ability of the COI and its dimensions to discriminate between the two groups of dogs, while the LOAD showed an excellent ability to do so. Although we included a relatively large sample, a broader sample would be preferred for future studies. Considering these results, the previously proposed working MCID of -4 for LOAD [20] seems appropriate for dogs with OA. This value is supported by the ROC (-2.5) anchor-based method and both distribution-based methods (ES ±1.6 and SEM ±4.2). Support for the previously proposed working MCID of -14 for the COI [20] is also reasonable, based on MDC (-14.5), ROC (-4.5), ES (±2.4), and SEM (±2.3) methods. For the different dimensions of the COI, a working MCID of -4 for Stiffness, Function, and Gait can be proposed, and of -3 for the quality of life, based on the different methods. These results reinforce the previously proposed estimates for use in sample size calculation and as a reference for researchers and regulators.

As with different evaluation modalities, and as a proxy completes the CROMs, the risk for a caregiver placebo effect exists. This effect has been attributed to the wish for the dog to get better, but also, in the case of OA, to the fact that some animals may exhibit some level of improvement due to the disease’s natural evolution and a regression to the mean effect [35–38]. The LOAD has not shown a major placebo effect in previous reports. This finding has been attributed to the emphasis of the questions on activity “activity/exercise” and “stiffness/lameness [13]. This rationale has been supported by a placebo effect not being found at the animal level, as the ability to perform daily activities will likely reflect a lower level of pain and disease impairment [39, 40]. Similar to the LOAD, much of the emphasis of the COI’s question is placed on the animal’s ability to perform daily activities. Criterion validity versus objective assessment of load-bearing has been observed for the LOAD and the COI [16]. Still, the scores observed in the “the same” group had a very small variation, in contrast to what was observed in the “somewhat better” group, consistent with the anchor question. With that in mind, while a certain level of caregiver placebo effect could be present in the present data, particularly since some treatments were more invasive in nature [41], it should not have influenced the results significantly. Future should include an objective measure, that allows the comparison of objective data with the results of the CROM.

This study had some limitations. The data were obtained from a specific population of dogs, and future studies should focus on a broader sample. Only patients with hip OA were included, and OA from other joints should also be considered. Different post treatment follow-up moments should also be considered, to determine if this factor influences the MCID. Future studies should also look at determining what would constitute a client-acceptable clinical state, a threshold where a client is likely to define the outcome as “satisfactory”, and the substantial clinical benefit level, defined as the clinical value that the client considers as “substantial improvement” [20].

## Conclusions

We presented estimates of MCIDs for LOAD and COI in dogs with OA, consistent with previously proposed values of -4 and -14, respectively. For the first time, we also presented estimates for the dimensions of the COI, of -4 for Stiffness, Function, and Gait can be proposed, and of -3 for quality of life. These estimates can be used for research and patient monitoring. Future studies should include OA from other joints and animals from a broader clinical context.

## References

[1] Anderson KL, Zulch H, O’Neill DG, Meeson RL, Collins LM. Risk Factors for Canine Osteoarthritis and Its Predisposing Arthropathies: A Systematic Review. Front Vet Sci. 2020;7. doi: 10.3389/fvets.2020.00220 32411739 PMC7198754

[2] Alves JC, Santos A, Jorge P, Lavrador C, Carreira LM. Clinical and diagnostic imaging findings in police working dogs referred for hip osteoarthritis. BMC Vet Res. 2020;16: 425. doi: 10.1186/s12917-020-02647-2 33160336 PMC7648415

[3] Anderson KL, O’Neill DG, Brodbelt DC, Church DB, Meeson RL, Sargan D, et al. Prevalence, duration and risk factors for appendicular osteoarthritis in a UK dog population under primary veterinary care. Sci Rep. 2018;8: 5641. doi: 10.1038/s41598-018-23940-z 29618832 PMC5884849

[4] Alves JC, Santos A, Jorge P, Lavrador C, Carreira LM. Characterization of Weight-bearing Compensation in Dogs With Bilateral Hip Osteoarthritis. Top Companion Anim Med. 2022;49: 100655. doi: 10.1016/j.tcam.2022.100655 35272058

[5] Clough W, Canapp S, Taboada L, Dycus D, Leasure C. Sensitivity and specificity of a weight distribution platform for the detection of objective lameness and orthopaedic disease. Vet Comp Orthop Traumatol. 2018;31: 391–395. doi: 10.1055/s-0038-1667063 30300913

[6] Meeson RL, Todhunter RJ, Blunn G, Nuki G, Pitsillides AA. Spontaneous dog osteoarthritis—a One Medicine vision. Nat Rev Rheumatol. 2019. doi: 10.1038/s41584-019-0202-1 30953036 PMC7097182

[7] Gruen ME, Griffith EH, Thomson AE, Simpson W, Lascelles BDX. Criterion Validation Testing of Clinical Metrology Instruments for Measuring Degenerative Joint Disease Associated Mobility Impairment in Cats. Thamm D, editor. PLoS One. 2015;10: e0131839. doi: 10.1371/journal.pone.0131839 26162101 PMC4498683

[8] Reid J, Nolan AM, Scott EM. Measuring pain in dogs and cats using structured behavioural observation. Vet J. 2018;236: 72–79. doi: 10.1016/j.tvjl.2018.04.013 29871754

[9] Alves JCA, Jorge PIF, dos Santos AMMP. A survey on the orthopedic and functional assessment in a Portuguese population of police working dogs. BMC Vet Res. 2022;18: 116. doi: 10.1186/s12917-022-03221-8 35337332 PMC8952961

[10] Albuquerque N, Guo K, Wilkinson A, Savalli C, Otta E, Mills D. Dogs recognize dog and human emotions. Biol Lett. 2016;12: 20150883. doi: 10.1098/rsbl.2015.0883 26763220 PMC4785927

[11] Wiseman-Orr ML, Nolan AM, Reid J, Scott EM. Development of a questionnaire to measure the effects of chronic pain on health-related quality of life in dogs. Am J Vet Res. 2004;65: 1077–1084. doi: 10.2460/ajvr.2004.65.1077 15334841

[12] Walton B, Cox T, Innes J. ‘How do I know my animal got better?’–measuring outcomes in small animal orthopaedics. In Pract. 2018;40: 42–50. doi: 10.1136/inp.k647

[13] Walton MB, Cowderoy E, Lascelles D, Innes JF. Evaluation of construct and criterion validity for the ‘Liverpool Osteoarthritis in Dogs’ (LOAD) clinical metrology instrument and comparison to two other instruments. Wade C, editor. PLoS One. 2013;8: e58125. doi: 10.1371/journal.pone.0058125 23505459 PMC3591443

[14] Brown DC. The Canine Orthopedic Index. Step 2: Psychometric testing. Vet Surg. 2014;43: 241–246. doi: 10.1111/j.1532-950X.2014.12141.x 24512284

[15] Alves JC, Jorge P, Santos A. Initial psychometric evaluation of the Portuguese version of the Liverpool Osteoarthritis in Dogs. BMC Vet Res. 2022;18: 367. doi: 10.1186/s12917-022-03461-8 36203166 PMC9535868

[16] Alves JC, Santos A, Jorge P, Lavrador C, Carreira LM. Evaluation of Four Clinical Metrology Instruments for the Assessment of Osteoarthritis in Dogs. Animals. 2022;12: 2808. doi: 10.3390/ani12202808 36290195 PMC9597733

[17] Radke H, Joeris A, Chen M. Evidence‐based evaluation of owner‐reported outcome measures for canine orthopedic care–a COSMIN evaluation of 6 instruments. Vet Surg. 2022;51: 244–253. doi: 10.1111/vsu.13753 34958495

[18] Monteiro BP, Lascelles BDX, Murrell J, Robertson S, Steagall PVM, Wright B. 2022 WSAVA guidelines for the recognition, assessment and treatment of pain. J Small Anim Pract. 2023;64: 177–254. doi: 10.1111/jsap.13566

[19] Brown DC, Bell M, Rhodes L. Power of treatment success definitions when the Canine Brief Pain Inventory is used to evaluate carprofen treatment for the control of pain and inflammation in dogs with osteoarthritis. Am J Vet Res. 2013;74: 1467–1473. doi: 10.2460/ajvr.74.12.1467 24274882

[20] Innes JF, Morton MA, Lascelles BDX. Minimal clinically-important differences for the ‘Liverpool Osteoarthritis in Dogs’ (LOAD) and the ‘Canine Orthopedic Index’ (COI) client-reported outcomes measures. Evans R, editor. PLoS One. 2023;18: e0280912. doi: 10.1371/journal.pone.0280912 36730152 PMC9894389

[21] Sedaghat AR. Understanding the Minimal Clinically Important Difference (MCID) of Patient‐Reported Outcome Measures. Otolaryngol Neck Surg. 2019;161: 551–560. doi: 10.1177/0194599819852604 31159641

[22] Alves JC. Initial Psychometric Evaluation of the Portuguese Version of the Canine Orthopedic Index. Vet Comp Orthop Traumatol. 2023. doi: 10.1055/s-0043-1768231 37160258

[23] Hercock CA, Pinchbeck G, Giejda A, Clegg PD, Innes JF. Validation of a client-based clinical metrology instrument for the evaluation of canine elbow osteoarthritis. J Small Anim Pract. 2009;50: 266–271. doi: 10.1111/j.1748-5827.2009.00765.x 19527419

[24] Alves JC, Santos A, Jorge P, Lavrador C, Carreira LM. Comparison of clinical and radiographic signs of hip osteoarthritis in contralateral hip joints of fifty working dogs. Banzato T, editor. PLoS One. 2021;16: e0248767. doi: 10.1371/journal.pone.0248767 33735210 PMC7971486

[25] Alves JC, Santos A, Jorge P, Carreira LM. A randomized double-blinded controlled trial on the effects of photobiomodulation therapy in dogs with osteoarthritis. Am J Vet Res. 2022;83. doi: 10.2460/ajvr.22.03.0036 35895799

[26] Alves JC, Santos A, Jorge P, Lafuente P. A multiple-session mesotherapy protocol for the management of hip osteoarthritis in police working dogs. Am J Vet Res. 2022; 1–8. doi: 10.2460/ajvr.22.08.0132 36367787

[27] Alves JC, Santos A, Jorge P. Platelet-rich plasma therapy in dogs with bilateral hip osteoarthritis. BMC Vet Res. 2021;17: 207. doi: 10.1186/s12917-021-02913-x 34090433 PMC8180029

[28] Alves JCC, Santos A, Jorge P, Lavrador C, Carreira LMM. Intraarticular triamcinolone hexacetonide, stanozolol, Hylan G-F 20 and platelet concentrate in a naturally occurring canine osteoarthritis model. Sci Rep. 2021;11: 3118. doi: 10.1038/s41598-021-82795-z 33542412 PMC7862601

[29] Alves JCA, Santos AMMP dos, Jorge PIF, Lavrador CFTVB, LMA Carreira. Management of Osteoarthritis Using 1 Intra-articular Platelet Concentrate Administration in a Canine Osteoarthritis Model. Am J Sports Med. 2021;49: 599–608. doi: 10.1177/0363546520981558 33428459

[30] Alves JCJC, Santos A, Jorge P, Lavrador C, Carreira LM, Miguel Carreira L. Correction: The intra-articular administration of triamcinolone hexacetonide in the treatment of osteoarthritis. Its effects in a naturally occurring canine osteoarthritis model. PLoS One. 2021;16: e0248082. doi: 10.1371/journal.pone.0248082 33635896 PMC7909660

[31] Alves JC, Santos A, Jorge P, Lavrador C, Carreira LM. Effect of a single intra-articular administration of stanozolol in a naturally occurring canine osteoarthritis model: a randomised trial. Sci Rep. 2022;12: 5887. doi: 10.1038/s41598-022-09934-y 35393497 PMC8989994

[32] Coretti S, Ruggeri M, McNamee P. The minimum clinically important difference for EQ-5D index: a critical review. Expert Rev Pharmacoecon Outcomes Res. 2014;14: 221–233. doi: 10.1586/14737167.2014.894462 24625040

[33] Revicki D, Hays RD, Cella D, Sloan J. Recommended methods for determining responsiveness and minimally important differences for patient-reported outcomes. J Clin Epidemiol. 2008;61: 102–109. doi: 10.1016/j.jclinepi.2007.03.012 18177782

[34] Ogura T, Ackermann J, Barbieri Mestriner A, Merkely G, Gomoll AH. Minimal Clinically Important Differences and Substantial Clinical Benefit in Patient-Reported Outcome Measures after Autologous Chondrocyte Implantation. Cartilage. 2020;11: 412–422. doi: 10.1177/1947603518799839 30221977 PMC7488950

[35] Brown DC. The Canine Orthopedic Index. Step 3: Responsiveness Testing. Vet Surg. 2014;43: 247–254. doi: 10.1111/j.1532-950X.2014.12162.x 24617818

[36] Vasseur PB, Johnson AL, Budsberg SC, Lincoln JD, Toombs JP, Whitehair JG, et al. Randomized, controlled trial of the efficacy of carprofen, a nonsteroidal anti-inflammatory drug, in the treatment of osteoarthritis in dogs. J Am Vet Med Assoc. 1995;206: 807–11. Available: http://www.ncbi.nlm.nih.gov/pubmed/7759332 7759332

[37] Brown DC, Boston RC, Coyne JC, Farrar JT. Ability of the canine brief pain inventory to detect response to treatment in dogs with osteoarthritis. J Am Vet Med Assoc. 2008;233: 1278–83. Available: http://www.ncbi.nlm.nih.gov/pubmed/19180716 doi: 10.2460/javma.233.8.1278 19180716 PMC2896492

[38] Dobenecker B, Beetz Y, Kienzle E. A Placebo-Controlled Double-Blind Study on the Effect of Nutraceuticals (Chondroitin Sulfate and Mussel Extract) in Dogs with Joint Diseases as Perceived by Their Owners. J Nutr. 2002;132: 1690S–1691S. doi: 10.1093/jn/132.6.1690S 12042495

[39] Conzemius MG, Evans RB. Caregiver placebo effect for dogs with lameness from osteoarthritis. J Am Vet Med Assoc. 2012;241: 1314–9. doi: 10.2460/javma.241.10.1314 23113523

[40] Piel MJ, Kroin JS, Van Wijnen AJ, Kc R, Im HJ. Pain assessment in animal models of osteoarthritis. Gene. 2014;537: 184–188. doi: 10.1016/j.gene.2013.11.091 24333346 PMC3950312

[41] Hróbjartsson A, Emanuelsson F, Skou Thomsen AS, Hilden J, Brorson S. Bias due to lack of patient blinding in clinical trials. A systematic review of trials randomizing patients to blind and nonblind sub-studies. Int J Epidemiol. 2014;43: 1272–1283. doi: 10.1093/ije/dyu115 24881045 PMC4258786

